# Artificial Intelligence in Dementia: A Bibliometric Study

**DOI:** 10.3390/diagnostics13122109

**Published:** 2023-06-19

**Authors:** Chieh-Chen Wu, Chun-Hsien Su, Md. Mohaimenul Islam, Mao-Hung Liao

**Affiliations:** 1 Department of Healthcare Information and Management, School of Health Technology, Ming Chuan University, Taipei 333, Taiwan; drluiswu@gmail.com; 2 Department of Exercise and Health Promotion, College of Kinesiology and Health, Chinese Culture University, Taipei 111369, Taiwan; chsu@ulive.pccu.edu.tw; 3 Graduate Institute of Sports Coaching Science, College of Kinesiology and Health, Chinese Culture University, Taipei 11114, Taiwan; 4 College of Pharmacy, The Ohio State University, Columbus, OH 43210, USA; d610106004@tmu.edu.tw; 5 Superintendent Office, Yonghe Cardinal Tien Hospital, New Taipei City 23148, Taiwan; 6 Department of Healthcare Administration, Asia Eastern University of Science and Technology, Banciao District, New Taipei City 220303, Taiwan

**Keywords:** artificial intelligence, Alzheimer’s disease, dementia, deep learning, machine learning

## Abstract

The applications of artificial intelligence (AI) in dementia research have garnered significant attention, prompting the planning of various research endeavors in current and future studies. The objective of this study is to provide a comprehensive overview of the research landscape regarding AI and dementia within scholarly publications and to suggest further studies for this emerging research field. A search was conducted in the Web of Science database to collect all relevant and highly cited articles on AI-related dementia research published in English until 16 May 2023. Utilizing bibliometric indicators, a search strategy was developed to assess the eligibility of titles, utilizing abstracts and full texts as necessary. The Bibliometrix tool, a statistical package in R, was used to produce and visualize networks depicting the co-occurrence of authors, research institutions, countries, citations, and keywords. We obtained a total of 1094 relevant articles published between 1997 and 2023. The number of annual publications demonstrated an increasing trend over the past 27 years. *Journal of Alzheimer’s Disease* (39/1094, 3.56%), *Frontiers in Aging Neuroscience* (38/1094, 3.47%), and *Scientific Reports* (26/1094, 2.37%) were the most common journals for this domain. The United States (283/1094, 25.86%), China (222/1094, 20.29%), India (150/1094, 13.71%), and England (96/1094, 8.77%) were the most productive countries of origin. In terms of institutions, Boston University, Columbia University, and the University of Granada demonstrated the highest productivity. As for author contributions, Gorriz JM, Ramirez J, and Salas-Gonzalez D were the most active researchers. While the initial period saw a relatively low number of articles focusing on AI applications for dementia, there has been a noticeable upsurge in research within this domain in recent years (2018–2023). The present analysis sheds light on the key contributors in terms of researchers, institutions, countries, and trending topics that have propelled the advancement of AI in dementia research. These findings collectively underscore that the integration of AI with conventional treatment approaches enhances the effectiveness of dementia diagnosis, prediction, classification, and monitoring of treatment progress.

## 1. Introduction

Currently, the use of artificial intelligence (AI) has reached widespread popularity at an unprecedented rate [1]. AI algorithms have emerged as potential tools in diverse areas of healthcare, including chronic disease management and clinical decision-making [2,3,4]. AI is playing a prominent role in dementia research due to advancements in computing power, novel algorithms, and the availability of big data generated from medical health records and wearable devices [5,6,7]. Recent studies in this domain have primarily focused on developing effective tools to accurately and quickly diagnose dementia, investigate the progression of dementia symptoms, and improve care and support for individuals affected by dementia [8,9].

The high number of continuously published articles and the rapid progress of AI research in the field of dementia has created significant challenges when it comes to staying up-to-date. Consequently, it becomes crucial to grasp the applications, significance, trends, and research hotspots within AI research in dementia. Systematic reviews, meta-analyses, and bibliometric analyses are employed to assist authors, clinicians, and policymakers in staying updated with emerging scientific outcomes [10,11,12]. Among these methods, bibliometric analysis is particularly valuable for the quantitative assessment of scientific literature, enabling the identification of key themes and emerging trends within specific research topics [13,14,15]. Bibliometric analyses always offer valuable insights by scrutinizing citations, co-citations, geographical distribution, and word frequency of literature.

The primary objective of this study was to provide a comprehensive overview of AI research in the field of dementia while also identifying future research directions that can benefit the general population, healthcare policymakers, and researchers. To address these goals, this study formulated the following research questions:

RQ1: What are the fundamental characteristics of the published articles? How many articles focusing on AI applications for dementia have been published to date?

RQ2: Who are the most productive authors/co-authors in these areas, and what are their countries of origin?

RQ3: Which journal has published the highest number of articles in this field? Which organizations have made significant contributions to this area of research?

RQ4: What are the most commonly used keywords associated with these publications?

By addressing these research questions, a comprehensive understanding of the current state of AI research in dementia can be obtained, enabling insights into the key contributors, research trends, and impactful literature in this domain.

## 2. Methods

### 2.1. Data Sources and Search Strategy

We conducted an extensive search to collect relevant publications in the Web of Science (WoS) related to AI applications in the area of dementia. This study only used WoS to retrieve articles due to its comprehensive collection of multidisciplinary academic journals and its status as an authoritative source for citation information, making it a primary scientific database for many researchers. WoS encompasses various sources, including Science Citation Index Expanded, Social Sciences Citation Index, Arts & Humanities Citation Index, Conference Proceedings Citation Index—Science, Conference Proceedings Citation Index—Social Science & Humanities, Emerging Sources Citation Index, and Current Chemical Reactions [16]. WoS has gained substantial recognition and has become a reliable source for conducting bibliometric analyses [1,17,18]. This is mainly attributed to its inclusion of key characteristics used for bibliometric studies, such as title, author, institution, country/region, publication year, and keywords [19]. A full search strategy is provided in Supplementary Table S1.

### 2.2. Inclusion and Exclusion Criteria

For this study, all articles that focused on the application of AI in the area of dementia were included for screening. The following inclusion criteria were applied: (1) articles written in the English language and (2) articles that focused on AI in dementia research. Given that AI is a rapidly evolving and dynamic research field, this study incorporated various types of articles, such as research or review articles published in peer-reviewed journals, conference proceedings, reviews, and early access articles. However, to maintain the focus and coherence of the bibliometric analysis, letters, editorials, book chapters, and books were excluded from the selection process.

### 2.3. Data Collection and Preprocessing

To ensure compatibility with Bibliometrix [20], all articles were saved using the name in the format download_**.txt. This file format is recognized by Bioliometrix and facilitates subsequent data processing. All valuable information from the articles was saved in documents, including titles, authors, countries, institutions, abstracts, keywords, journals, and publication dates.

### 2.4. Bibliometric Analysis

We employed Bibliometrix, a statistical software tool within the R environment. The preprocessed data were imported into Bibliometrix, and bibliometric analysis was performed based on the information contained in the data documents. Our objective was to present a comprehensive overview of AI research in the field of dementia, with a focus on elucidating the knowledge structure, identifying influential authors, uncovering research trends, finding the most productive countries and institutions, and identifying research hotspots. We calculated the total number of publications per year, yearly growth rate, and average growth rate of publications per year; however, the following methods were employed to calculate annual growth and average growth rate:

$$\begin{array}{c} Annual\;growth\\ \;=\;(Total\;number\;of\;publications\;in\;current\;year\\ \;-\;Total\;number\;of\;publications\;in\;the\;previous\;year) \end{array}$$

$$Average\;growth\;rate\left(AGR\right)=\frac{\left[\left(P-P_{n-1}\right)\right]}{P_{n-1}}\times 100$$

where P is the total number of publications in the current year, and $P_{n-1}$ is the total number of publications in the previous year.

In this study, we conducted an analysis of publication trends based on the following factors: the top 10 most productive countries, source journals, the distribution and co-authorship of institutions, and authors. The rankings for countries, journals, institutions, and authors were determined based on the number of articles they published.

## 3. Results

### 3.1. Publication Output

**Figure 1:**
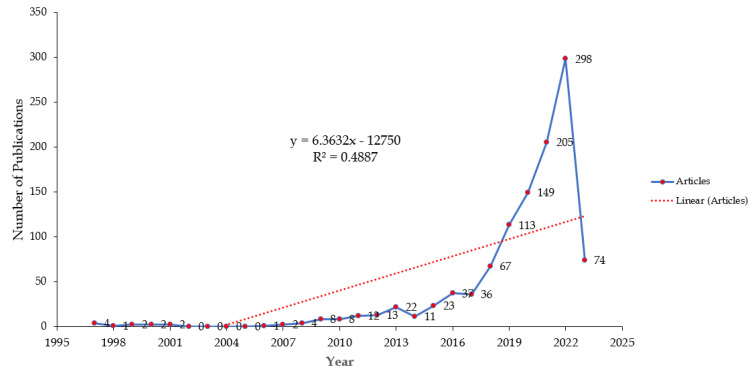
Number of AI-related research in the area of dementia from 1997 to 2023.

Based on our extensive search on WoS, we identified a total of 1236 articles on AI research in the area of dementia. After excluding 142 articles based on predetermined criteria, a total of 1094 articles were included for analysis (as shown in Figure 1). The number of publications in this area has exhibited a significant growth trend over the years. Notably, the annual publication count has risen from 4 articles in 1997 to 298 articles in 2022. It is worth mentioning that prior to 2018, the annual publication count did not surpass 100 articles.

### 3.2. Distribution of Source Journals

A total of 1094 journals published articles on AI research in the field of dementia, with 848 of these journals contributing only a single paper on mobile health apps. Table 1 presents the top 10 journals in terms of the number of publications in this area. Among these, the *Journal of Alzheimer’s Disease* published the highest number of articles (39/1094, 3.56%), followed by *Frontiers in Aging Neuroscience* (38/1094, 3.47%) and *Scientific Reports* (26/1094, 2.37%). Collectively, these top 10 journals accounted for 198 articles, representing 18.09% of all publications included in this study.

Table 2 shows the location of the top 10 countries that published AI-related research on dementia. The People’s Republic of China had the highest number of publications, with 214, and the USA ranked second with 143, followed by India and South Korea. However, the ratio of multiple country publications (MCP) was higher in the United Kingdom (28/59, 0.47), followed by Saudi Arabia (11/23, 0.47%).

Based on the search results, a total of 1943 institutions contributed to AI research in the area of dementia. Table 3 displays the top 10 most prolific institutions in this domain. Boston University (49 publications) ranked first among all institutions identified, followed by Columbia University (41 publications) and the University of Granada (36 publications).

**Figure 2:**
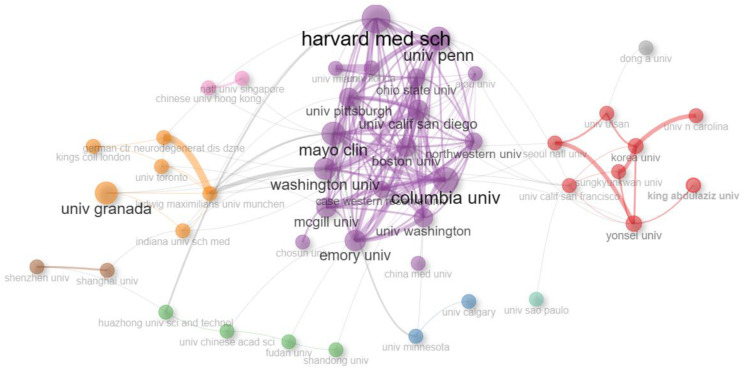
The co-authorship network of institutions that contributed to AI research on dementia, 1997–2023.

A co-authorship analysis using Bibliomatrix was conducted to generate a visual network map of institutions involved in AI research on dementia. The connections between institutions were established based on the number of co-authored articles published between them. The co-authorship analysis revealed that 45 institutions, each contributing at least five papers, formed nine distinct clusters. Figure 2 illustrates these clusters, which are denoted by different colors, providing a visual representation of the collaborative network among institutions in this research field.

In Table 4, we present the 10 leading authors who actively conducted research and published articles in the field of dementia. A total of 5360 authors contributed 1094 publications on this subject. Gorriz JM holds the top position, with 27 articles, closely followed by Ramirez J, with the same number of publications. Salas-Gonzalez D ranks third, with 17 articles, while Segovia F follows closely, with 16 articles. Notably, it is worth mentioning that all four of these top authors are affiliated with the same institution.

By conducting a co-citation analysis, we aimed to establish a knowledge base for AI research in the field of dementia. In this analysis, we included a total of 1094 publications focusing on AI-related aspects of dementia, which collectively cited 31,085 references. On average, each publication cited 16.11 references. Table 5 presents the top 10 most frequently cited references in this analysis. The publication that received the highest number of citations was “*Machine learning framework for early MRI-based Alzheimer’s conversion prediction in MCI subjects,*” by Moradi E. and colleagues, which was published in *Neuroimage* in 2015. As of 15 May 2023, this publication accumulated a total of 402 citations.

In addition to analyzing the research subjects, we explored the keywords utilized by the authors, which serve as indicators of the primary research themes covered in the publications. A total of 2109 keywords were identified across 1094 articles. Table 6 displays the prominent keywords found in different subgroups, including disease classification, models, algorithms, modalities, and outcomes. Out of the 1094 articles, 513 featured Alzheimer’s disease as the primary keyword. Notably, convolutional neural networks, support vector machines, and random forest algorithms were the most frequently mentioned keywords among the retrieved articles.

**Figure 3:**
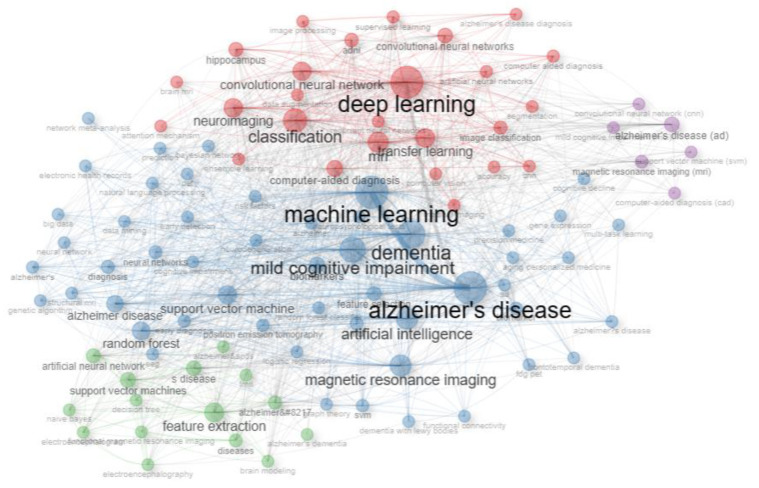
The co-occurrence network of the top 100 keywords in AI research for dementia, 1997–2023.

To identify the research hot spots within AI-related research on dementia, a co-occurrence analysis of the top 100 keywords was conducted. The Bibliometrix tool was employed to extract and cluster these keywords, unveiling patterns of clustering. Figure 3 visually illustrates the clustering patterns of these top 100 keywords, providing insights into the prominent research areas and themes within AI research on dementia.

## 4. Discussion

This bibliometric analysis aimed to present a comprehensive overview of research on AI-related research in the field of dementia. The analyses encompassed a total of 1094 publications spanning 27 years, from 1997 to the partial year of 2023. The key findings of this study are as follows:

(a) The research publications related to AI in dementia have exhibited a notable growth trend over time; 

(b) Developed countries have emerged as the primary as primary drivers of AI research in dementia care. However, due to the increasing aging population, countries worldwide are actively engaged in dementia research;

(c) The majority of highly cited researchers in this area are affiliated with prestigious universities; 

(d) Collaboration among universities in the USA demonstrated the highest level, followed by South Korean universities. 

Additionally, this study identified the most prevalent research categories, popular keywords, and critical terms within the AI-related research on dementia. These findings underscore the importance of further exploration in this field to enhance AI-based research for managing dementia. By providing a comprehensive summary of the AI research trends in dementia, this work aims to serve as a guiding resource for future research endeavors, fostering advancements in this particular field of study.

The advent of AI research has presented exciting possibilities for the development of efficient and accessible tools that can aid in the early prediction of dementia [21]. Previous evidence has demonstrated the potential of AI to assist physicians in conducting specific tests and investigations for more effective management of dementia [22,23,24]. Our study findings show that the majority of research has focused on various aspects of dementia, including disease classification, diagnosis, prediction, segmentation, and early detection. Several studies have also highlighted the association between healthy aging and certain types of cognitive decline, such as processing speed, fluid reasoning, and episodic memory [25,26,27]; however, it is worth noting that around 5–15% of individuals eventually develop dementia [28]. Globally, over 50 million people are currently living with dementia, and the number is projected to rise due to the increasing aging population [29]. Since there are no specific treatment options for dementia, the development of automated tools for the earliest possible detection is crucial in maximizing the impact of existing and traditional treatment approaches to delay pathological cognitive aging [30]. 

The area of AI in dementia research has garnered global attention as dementia has emerged as a significant public health concern [31]. While high-income countries have been the primary drivers of AI research in dementia, low- and middle-income countries are also increasingly focusing on this area. However, the output of research from low- and middle-income countries remains comparatively lower than that of high-income countries due to limited resources and technological advancements. In recent times, several developed countries have implemented AI healthcare policies that provide guidance on the development and regulation of AI in healthcare, such as the UK Code of Conduct for Data-Driven Health and Care Technology [32]. It is crucial to encourage researchers from low- and middle-income countries to engage in AI research by offering funding, research opportunities, and access to tools. AI-based automated tools hold promise in improving health outcomes, particularly in low-income countries with limited healthcare resources [33,34,35].

AI research in dementia is predominantly favored by prominent healthcare journals, as indicated by the output and citation counts. The majority of these studies tend to be specific to the field of neurological disorders. Nevertheless, AI-related research also attracts attention from computer science and multidisciplinary journals. It is important to note that open access journals and those with high impact factors tend to receive a greater number of citations [36,37]. This can be attributed to the perceived quality and reliability of research published in high-impact-factor journals, as well as the increased visibility of articles in open access journals. Over the past few decades, the occurrence and duration of citation bursts related to AI research on dementia have displayed variation. Certain keywords have consistently remained significant over an extended period, indicating the enduring focus of dementia research. As indicated by the keywords provided by the authors, convolutional neural networks, support vector machines, and random forest models have made a substantial impact on dementia research. These techniques have been widely used in dementia studies for classification, diagnosis, early prediction, and detection [38,39,40]. The field of AI research in dementia has witnessed significant paradigm shifts over the years and continues to evolve. Dementia research exerts strong impacts on healthcare, and our study findings suggest that the impact of AI in dementia research is likely to persist in the coming years.

Over the past few decades, the occurrence and duration of citation bursts related to AI research on dementia have displayed variations. Certain keywords have consistently remained significant over an extended period, indicating the enduring focus of dementia research. As indicated by the keywords provided by the authors, convolutional neural networks, support vector machines, and random forest models have made a substantial impact on dementia research. These techniques have been widely utilized in dementia studies for classification, diagnosis, early prediction, and detection [38,39,40]. The field of AI research in dementia has witnessed significant paradigm shifts over the years and continues to evolve. These research domains exert a strong influence on healthcare, and our study findings suggest that the impact of AI in dementia research is likely to persist in the coming years.

To the best of our knowledge, this study represents the first bibliometric analysis of AI-based research on dementia. However, it is important to acknowledge the limitations of this study. First, there might be a language bias as we focused solely on publications in English. Second, despite our efforts to capture all articles on AI-based research in dementia over the past 27 years, certain sources like gray literature and reports from databases other than WoS may have been omitted. The number of citations reported in this study is based on WoS records and may differ from those in Google Scholar. Additionally, more recent articles are likely to accumulate additional citations in the future, potentially resulting in the exclusion of high-quality studies from our analysis. Lastly, the data used for analysis was solely extracted from WoS, excluding databases such as Scopus (Elsevier) and PubMed. Although WoS includes high-quality journals, there is a possibility that some articles may have been missed.

## 5. Conclusions

This study sheds light on potential emerging trends in AI research on dementia through a comprehensive bibliometric analysis. This study reveals the most prolific countries, institutions, authors, journals, research trends, and hot spots in this domain by examining publication outputs spanning the years 1997 to 2023. The findings presented in this study hold valuable insights that can guide clinical decision-making and inform future research endeavors in the field of AI and dementia.

## Figures and Tables

**Table 1:** Top 10 journals publishing research on AI-related research in the area of dementia, 1997–2023.

Rank	Journal	Country	Category	Publication, n	Percentage of Article	Impact Factor in 2021	5-Year Impact Factor
1	*Journal of Alzheimer’s Disease*	Netherlands	Neurosciences	39	3.56	4.16	5.27
2	*Frontiers in Aging Neuroscience*	Switzerland	Neurosciences	38	3.47	5.70	6.22
3	*Scientific Reports*	England	Multidisciplinary Science	26	2.37	4.99	5.51
4	*PLoS ONE*	USA	Multidisciplinary Science	24	2.19	3.75	4.06
5	*IEEE Access*	USA	Computer science, Information system	15	1.37	3.47	3.75
6	*Frontiers in Neuroscience*	Switzerland	Neurosciences	12	1.09	5.15	5.58
7	*Alzheimer’s Research and Therapy*	England	Neurosciences	11	1.00	8.83	9.03
8	*Applied Sciences-Basel*	Switzerland	Engineering, Multidisciplinary	11	1.00	2.83	2.91
9	*Computers in Biology and Medicine*	USA	Computer Science, Interdisciplinary Applications	11	1.00	6.69	5.75
10	*Diagnostics*	Switzerland	Medicine, General, Internal	11	1.00	3.99	4.12

Note: n = number of articles, country: origin of journals.

**Table 2:** Top 10 countries that published articles on artificial intelligence in the area of dementia, 1997–2023.

Rank	Country	Article	Frequency	SCP	MCP	MCP Ratio
1	China	214	0.19	152	62	0.29
2	USA	182	0.16	139	43	0.23
3	India	124	0.11	106	18	0.14
4	Korea	67	0.06	49	18	0.26
5	United Kingdom	59	0.05	31	28	0.47
6	Spain	54	0.04	45	9	0.16
7	Italy	52	0.04	31	21	0.40
8	Japan	36	0.03	28	8	0.22
9	Canada	30	0.02	21	9	0.30
10	Saudi Arabia	23	0.02	12	11	0.47

Abbreviation: Freq. = frequency; SCP = single country publication, MCP = multiple country publication (note: the countries were identified by the affiliations of the listed authors).

**Table 3:** Top 10 institutions that published articles on artificial intelligence in the area of dementia, 1997–2023.

Rank	Institutions	Country	Publications, (Percent)
1	Boston University	United States	49 (4.47)
2	Columbia University	United States	41 (3.74)
3	University of Granada	Spain	36 (3.29)
4	Seoul National University	South Korea	27 (2.49)
5	Washington University	United States	27 (2.46)
6	University of Pennsylvania	United States	25 (2.28)
7	Harvard Medical School	United States	24 (2.19)
8	Yonsei University	South Korea	23 (2.10)
9	Sungkyunkwan University	South Korea	22 (2.01)
10	University of Calgary	Canada	22 (2.01)

**Table 4:** Top 10 most productive authors published on artificial intelligence in the area of dementia, 1997–2023.

Rank	Author	Article	Citations	H-Index	Affiliation
1	Gorriz JM	27	6094	41	University of Granada
2	Ramirez J	27	5783	42	University of Granada
3	Salas-Gonzalez D	17	2333	30	University of Granada
4	Segovia F	16	2084	25	University of Granada
5	Li J	13	90	5	Nanjing Normal University
6	Wang L	13	3050	27	Northwestern University
7	Wang Y	11	602	11	Nanjing University
8	Lopez M	10	1596	20	University of Genoa
9	Alvarez I	9	2199	28	University of Granada
10	Chaves R	9	1241	19	Universidade de Sao Paulo

**Table 5:** Top 10 cited references in AI-related research in the area of dementia, 1997–2023.

Rank	Author	Journal	Title	Citation, n
1	Moradi E et al., 2015	*Neuroimage*	Machine learning framework for early MRI-based Alzheimer’s conversion prediction in MCI subjects	402
2	Liu SQ et al., 2015	*IEEE Transactions on Biomedical Engineering*	Multimodal Neuroimaging Feature Learning for Multiclass Diagnosis of Alzheimer’s Disease	301
3	Ding Y et al., 2019	*Radiology*	A Deep Learning Model to Predict a Diagnosis of Alzheimer Disease by Using F-18-FDG PET of the Brain	252
4	Shi J et al., 2018	*IEEE Journal of Biomedical and Health Informatics*	Multimodal Neuroimaging Feature Learning With Multimodal Stacked Deep Polynomial Networks for Diagnosis of Alzheimer’s Disease	234
5	Ortiz A et al., 2016	*International Journal of Neural System*	Ensembles of Deep Learning Architectures for the Early Diagnosis of the Alzheimer’s Disease	213
6	Jo T et al., 2016	*Frontiers in Aging Neuroscience*	Deep Learning in Alzheimer’s Disease: Diagnostic Classification and Prognostic Prediction Using Neuroimaging Data	194
7	Liu MH et al., 2020	*Neuroimage*	A multi-model deep convolutional neural network for automatic hippocampus segmentation and classification in Alzheimer’s disease	188
8	Spasov S et al., 2019	*Neuroimage*	A parameter-efficient deep learning approach to predict conversion from mild cognitive impairment to Alzheimer’s disease	169
9	Taveer M et al., 2020	*ACM Transactions on Multimedia Computing Communications and Applications*	Machine Learning Techniques for the Diagnosis of Alzheimer’s Disease: A Review	117
10	Ieracitano, C et al., 2020	*Neural Network*	A novel multi-modal machine learning based approach for automatic classification of EEG recordings in dementia	116

Note: citation information from WoS. The number of citations might vary if checked in Google Scholar.

**Table 6:** Top author keywords for artificial intelligence in the area of dementia research.

Keywords	Frequency (Percentage)
Disease classification	
Alzheimer’s disease	513 (46.89)
Dementia	164 (14.99)
Cognitive impairment	105 (9.59)
Model	
Machine Learning	297 (27.14)
Deep Learning	214 (19.56)
Algorithms	
Convolutional Neural Network	54 (4.93)
Support Vector Machine	40 (3.65)
Random Forest	36 (3.29)
Transfer Learning	30 (2.74)
Artificial Neural Network	17 (1.55)
Modality	
Magnetic Resonance Imaging	71 (6.48)
Neuroimaging	39 (3.56)
Biomarkers	21 (1.91)
Positron Emission Tomography	14 (1.27)
ECG	13 (1.18)
Tasks	
Classification	73 (6.67)
Diagnosis	24 (2.19)
Prediction	16 (1.46)
Segmentation	10 (0.91)
Early Detection	9 (0.82)

## Data Availability

Not applicable.

## Supplementary Materials

The following supporting information can be downloaded at: https://www.mdpi.com/article/10.3390/diagnostics13122109/s1, Table S1: Search keywords.
