# Supplementary material for: Artificial Intelligence in Dementia: A Bibliometric Study
Source: Diagnostics (Basel). 2023 Jun 19;13(12):2109. doi: 10.3390/diagnostics13122109 (PMC10297057; doi:10.3390/diagnostics13122109)
Supplement: Supplementary file 1 [file diagnostics-13-02109-s001.zip › diagnostics-2442181-supplementary.pdf]

Table S1: Search keywords

AI	artificial intelligence OR "computational intelligence" OR "deep learning" OR "computer aided" OR "machine learning" OR "support vector machine" OR "data learning" OR " artificial neural network" OR "digital image" OR "convolutional neural network" OR "evolutionary algorithms" OR "feature learning" OR "reinforcement learning" OR "big data" OR "image segmentation" OR "hybrid intelligent system" OR "hybrid intelligent system" OR "recurrent neural network" OR "natural language processing" OR " bayesian network " OR " bayesian learning" OR " random forest" OR "evolutionary algorithms" OR "multiagent system"
Dementia	Dementia OR Alzheimer's disease OR cognitive impairment
